# Mapping the reporting practices in recent randomised controlled trials published in *Knee Surgery, Sports Traumatology, Arthroscopy*: A scoping review of methodological quality

**DOI:** 10.1002/jeo2.70117

**Published:** 2025-01-07

**Authors:** Aleksandra Królikowska, Natalia Urban, Marcin Lech, Paweł Reichert, Nikolai Ramadanov, Mahmut Enes Kayaalp, Robert Prill

**Affiliations:** ^1^ Physiotherapy Research Laboratory University Centre of Physiotherapy and Rehabilitation, Faculty of Physiotherapy, Wroclaw Medical University Wroclaw Poland; ^2^ Evidence‐Based Healthcare in Wroclaw: A JBI Affiliated Group The University of Adelaide Adelaide South Australia Australia; ^3^ Clinical Department of Orthopedics, Traumatology and Hand Surgery Jan Mikulicz‐Radecki University Hospital Wroclaw Poland; ^4^ Department of Orthopedics, Traumatology and Hand Surgery Faculty of Medicine, Wroclaw Medical University Wroclaw Poland; ^5^ Center of Orthopaedics and Traumatology University Hospital Brandenburg/Havel, Brandenburg Medical School Theodor Fontane Brandenburg a.d.H. Germany; ^6^ Faculty of Health Sciences Brandenburg Brandenburg Medical School Theodor Fontane Brandenburg a.d.H. Germany; ^7^ Clinic of Orthopedics and Traumatology Istanbul Kartal Dr. Lutfi Kirdar Training and Research Hospital Istanbul Turkey

**Keywords:** adherence to guidelines, *ESSKA*, *KSSTA*, randomised controlled trial, risk of bias

## Abstract

The official medical journals of scientific societies advocate for high‐quality standards. It's important to assess whether randomized controlled trials (RCTs) in influential journals, such as the hybrid journal of the European Society of *Sports Traumatology, Knee Surgery*, and *Arthroscopy* (*ESSKA*), adhere to reporting guidelines and best practices. Therefore, the present scoping review aimed to explore and map the reporting practices and methodological quality in recent RCTs published in the *Knee Surgery, Sports Traumatology, Arthroscopy* (*KSSTA*) journal, focusing on identifying gaps in adherence to reporting guidelines and transparency. The study was preregistered and followed the PRISMA‐ScR checklist. RCTs published in *KSSTA* between 2022 and 2023 were included. The search was conducted via PubMed. A two‐stage selection process was employed, with two independent reviewers conducting study selection and data extraction. Data collected included study characteristics, intervention details, sample size calculation reporting, data transparency, and adherence to Consolidated Standards of Reporting Trials (CONSORT) guidelines. Critical appraisal was conducted using the JBI tool for RCTs. All included RCTs (*n* = 25) reported a predetermined minimum sample size. Study protocol preregistration was reported in 52% of the RCTs, while only 24% provided data availability statements. Most RCTs offering data availability indicated data would be shared upon request. Adherence to CONSORT guidelines was reported in 96% of studies, with only one RCT not adhering to recognized reporting standards. All the included studies adequately addressed statistical conclusion validity. However, internal validity was less consistently addressed across the studies.

**Conclusions:**

While most recently published RCTs in *KSSTA* adhered to CONSORT guidelines, there is potential for improvement in the reporting of protocol preregistration and data availability statements. Although all studies reported sample size calculations, transparency in data sharing remains limited.

**Level of Evidence:**

Level I.

**Abbreviations**
CONSORT: Consolidated Standards of Reporting Trials
*ESSKA*
: *European Society of Sports Traumatology, Knee Surgery and Arthroscopy*
*KSSTA*
: *Knee Surgery, Sports Traumatology, Arthroscopy*
MCID: minimal clinically important difference
PROM: patient‐reported outcome measure
RCT: randomised controlled trial
TKA: total knee arthroplasty

## BACKGROUND

The advancement of life sciences relies on training new generations of professionals who can enhance patient care through research. In orthopaedics, traumatology, and sports medicine, significant surgical innovations have emerged in recent decades. On the other hand, for example, in Europe, due to a lack of harmonisation and structural gaps in scientific training during and after medical education, specialists face challenges in gaining research competence, and some subspecialties like orthopaedic sports medicine are disappearing from academic centres, risking the ‘de‐academization’ of the scientific community [10]. Therefore, scientific societies like the *European Society of Sports Traumatology, Knee Surgery and Arthroscopy* (ESSKA) play a crucial role in advancing and sharing scientific knowledge and progress. They aim to enhance educational interaction, stimulate creative thinking and learning, exchange ideas, data, and content, promote research and facilitate scientific discussion and publication [10].

Based on that, the official medical journals of scientific societies play several important roles, including dissemination of knowledge and research, improvement of clinical practice and patient care, and promotion of education and professional development [51]. Also, their pivotal role in advocating for high‐quality standards should be noticed. By adhering to high research reporting standards and promoting relevant guidelines, journals advocate for methodological rigour and transparency [49]. This helps enhance the quality of research in the field and ensures that findings are reliable and applicable to clinical practice. Journals also reinforce the importance of ethical research practices, such as transparency, data availability, and reproducibility, which are essential for maintaining trust in medical research.

ESSKA consistently produces high‐quality educational and informative material through its two prominent online journals, one of which celebrated its 30th‐anniversary last year. The long‐established history and influence of *Knee Surgery, Sports Traumatology, Arthroscopy* (*KSSTA*) journal offer a valuable opportunity to explore and map the reporting practices and methodological quality in recent randomised controlled trials (RCTs), focusing on adherence to reporting guidelines and transparency.

Given the increasing volume of research published in high‐impact journals like the official journals of the *ESSKA*, there is a need to systematically explore whether RCTs published in those journals adhere to reporting guidelines and follow best practices in methodological rigour [9]. At the end of 2022, *KSSTA* initiated a series of articles focusing on improving evaluation standards for clinical studies in orthopaedics and sports medicine, including sample‐specific information, data transparency and adherence to reporting guidelines [49]. The question remains whether these three components on which good research starts are appropriately addressed or whether there are still gaps that need further clarification and emphasis.

It is important to examine how RCTs are reported and whether essential methodological details, such as sample size calculations and data transparency statements, are consistently included [47]. There also is a need to determine if RCTs adhere to established guidelines, such as the Consolidated Standards of Reporting Trials (CONSORT), to pinpoint areas where reporting can be enhanced [50]. By identifying shortcomings in reporting crucial methodological details, we can gain insights into areas where improvements are necessary for future research practices and editorial policies of thematic journals [9, 16].

Therefore, the present scoping review aimed to explore and map the reporting practices and methodological quality in recent RCTs published in *KSSTA* in 2022–2023.

It was hypothesised that most RCTs published in *KSSTA* between 2022 and 2023 adhere to key reporting guidelines, including CONSORT, and will adequately report essential methodological details such as a priori sample size calculations, protocol preregistration and data transparency statements.

## METHODS

The study was preregistered before data extraction with the Open Science Framework (https://doi.org/10.17605/OSF.IO/R7MH5). The present scoping review followed the Preferred Reporting Items for Systematic Reviews and Meta‐Analyses extension for Scoping Reviews Checklist when reporting its results [1].

### Eligibility criteria

The review included RCTs available as early online publications (e‐pub) in *KSSTA* from 2022 to 2023. Studies that were not identified as RCTs were excluded.

All articles in *KSSTA* are published in English. Therefore, specific language was not considered an inclusion or exclusion criterion. The review authors had full access to all early online publications, so full‐text availability is also not discussed.

### Information sources and search strategy

A search was performed using PubMed to identify all articles published in the journal *KSSTA* from 2022 to 2023. The search term was limited to the full name of the journal: ‘*Knee Surgery, Sports Traumatology, Arthroscopy: Official Journal of the ESSKA*’ [Journal]. Consecutively, the results were filtered by year to choose studies published in 2022–2024. The last search was carried out on 22 September 2024.

### Selection process

A two‐stage selection process was conducted. Two independent reviewers performed the study selection. Discrepancies were resolved through consultation with a third senior reviewer. All identified articles were initially screened by reviewing titles and abstracts to identify RCTs. Subsequently, the full texts of the indicated articles were reviewed to confirm they met the inclusion criteria.

### Data collection process

Two independent reviewers collected data from each included study. Each reviewer gathered relevant information and documented their findings using Microsoft Office Excel 365 Personal (Microsoft Corporation). The two reviewers compared their findings and resolved any discrepancies collaboratively. A senior reviewer was consulted if necessary for additional insights or to mediate any unresolved differences.

Since the review aimed to evaluate the quality of RCT reporting, no efforts were made to contact study investigators for further clarification or missing data. Only data explicitly reported in the published articles were included in the analysis.

### Data items

For each included study, the following data were sought and collected, focusing on how they were reported in the publications:

a) General data: authors; year of publication; title; country, where the study was performed.
b) Study‐specific data: primary health condition or problems studied; purpose of the study (treatment or prevention or diagnosis or other); description of intervention; comparator or control treatment; primary outcome(s) and assessment method; secondary outcome(s) and assessment method.
c) Sample‐specific information: number of participants (total and in consecutive groups); reporting minimal sample size calculation a priori (yes or no).
d) Data transparency: protocol preregistration reported (yes or no); the presence of a data availability statement (yes or no); if yes, specific information included.
e) Adherence to reporting guidelines: the presence of information about adhering to the CONSORT statement (yes or no) or any other recognised guidance for reporting RCTs.

### Critical appraisal of individual sources of evidence

Two independent reviewers manually assessed the risk of bias in the included studies using the revised JBI critical appraisal tool for RCT [2]. If there were identified discrepancies in the risk of bias ratings, the two independent reviewers initially discussed their assessments, explaining their interpretation of the criteria and justifying their ratings. If they could not reach a consensus through discussion, a third senior reviewer was consulted, reviewed the specific points of disagreement and made a final decision to resolve the discrepancy.

### Synthesis of results

In this scoping review, the synthesis of results focused on descriptive analysis rather than statistical or meta‐analytic techniques, given the goal of mapping and summarising reporting practices across the included RCTs.

For the synthesis, all included studies were systematically categorised based on key reporting elements, including general study characteristics: authors, year, country, study‐specific data: primary health condition or problems studied; purpose of the study (treatment or prevention or diagnosis or other); description of intervention; comparator or control treatment; primary outcome(s) and assessment method; secondary outcome(s) and assessment method; reporting quality indicators (sample‐specific information, data transparency, adherence to reporting guidelines). The results were presented as a descriptive analysis, summarising the overall trends in how RCTs reported their methodological details.

## RESULTS

**Figure 1:**
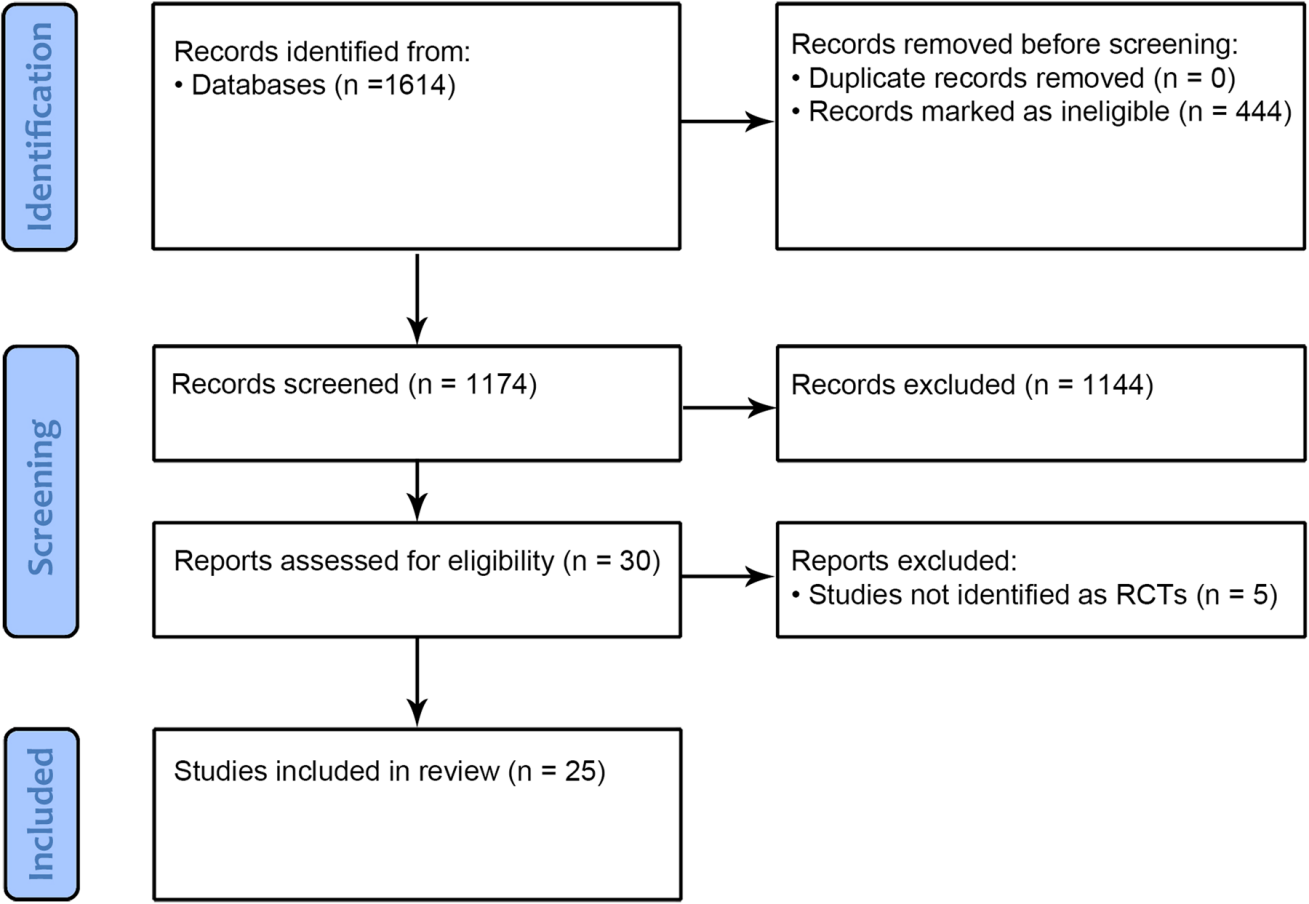
PRISMA flow diagram of the studies selection process. PRISMA, Preferred Reporting Items for Systematic Reviews and Meta‐Analyses.

The review included 25 RCTs [4, 5, 6, 11, 12, 14, 15, 17, 19, 29, 30, 32, 33, 37, 40, 46, 55, 56, 58, 59, 60, 61, 62, 66, 67]. The flowchart diagram of the literature search is shown in Figure 1.

General characteristics and study‐specific data of included RCTs were presented in detail in Table 1. In most included studies, the primary health condition or problem studied was total knee arthroplasty (TKA). In all studies, apart from one, the main purpose was treatment. Primary outcome(s) and secondary outcome(s) have been assessed using a wide variety of objective and subjective methods, including patient‐reported outcome measures (PROMs).

**Table 1:** General characteristics and study‐specific data of included studies.

General study characteristics		Study‐specific data					
Authors and year	Country	Primary health conditions or problems studied	Purpose of the study	Description of intervention	Comparator or control treatment	Primary outcome(s) and assessment method	Secondary outcome(s) and assessment method
Bollars et al. (2023)	Belgium	TKA	Treatment	Imageless handheld robotic‐assisted TKA	Active (conventional jig‐based instrumented TKA)	Radiologic measurements of achieved target hip–knee–ankle axis and implant component position including varus and external rotation and flexion of the femur component, and posterior tibial slope (CT scan)	None
Budhiparama et al. (2023)	Indonesia	TKA	Treatment	TKA using medial pivot implants and PCL preservation technique	Active (TKA using medial pivot implants and PCL resection technique)	ROM	Knee pain during walking (VAS) PROs (KOOS‐S, KOOS‐QoL, OKS, FJS) The measurement of the mechanical femoral‐tibial angle (radiographs)
Chu et al. (2022)	China	Knee osteoarthritis	Treatment	Pure platelet‐rich‐plasma injections	Sham (saline injections)	PROs (WOMAC)	PROs (IKDC) Pain (VAS) Intra‐articular biochemical marker concentrations Cartilage volume (MRI) Adverse events
Degen et al. (2023)	Canada	Femoroacetabular impingement syndrome	Treatment	Opioid medication	Active Opioid medication + postoperative sleeping aid Zopiclone Opioid medication + preoperative and postoperative Gabapentin Opioid medication + pre‐medicate with Celecoxib	Pain (VAS)	Opioid consumption Healthcare resource utilisation Length of hospital stay
Demirci et al. (2023)	Netherlands	Anxiety and pain in patients undergoing orthopaedic surgery	Treatment	Audio‐visual distraction with video glasses	Active (audio distraction)	Anxiety level (STAI‐6)	Pain (NRS) Vital signs Patient‐reported satisfaction
Farhan‐Alanie et al. (2023)	United Kingdom	TKA	Treatment	Navigated TKA	Active (conventional TKA)	Revision rates Mortality rates PROs (OKS, AKSS) ROM (hand‐held goniometer) Demographic statistics	Adverse events Levels of intra‐articular biochemical markers in synovial fluid post‐treatment Cartilage volume Pain (VAS)
Goicoechea et al. (2023)	Spain	TKA	Treatment	Lateral retinacular release	Active (no lateral retinacular release)	Pain (VAS)	None
Hoburg et al. (2022)	Germany, Poland	Knee cartilage defects	Treatment	Matrix‐associated autologous chondrocyte implantation using spheroids	Active (microfracture)	PROs (KOOS)	Magnetic resonance Cartilage repair tissue PROs (IKDC, Lysholm score)
Kalaai et al. (2023)	Netherlands, Belgium	TKA	Treatment	Deep‐dished mobile bearing cemented TKA	Active (fixed bearing cemented TKA)	Polyethylene wear rates PROs (KSS, OKS, WOMAC, LEAS, FJS‐12)	None
Lavoie et al. (2023)	Canada	TKA	Treatment	Bicruciate‐retaining prosthesis	Active (posterior‐stabilised prosthesis)	Patient‐reported outcomes (KSS, KOOS, WOMAC SF‐12) ROM	None
Lee et al. (2023)	South Korea	TKA (ERAS)	Treatment	Limited tourniquet	Active (conventional tourniquet)	Surgical time Haemoglobin drop Estimated blood loss Transfusion rates d‐dimer levels DVT incidence Pain (VAS) Opioid consumption ROM Knee circumference Postoperative complications Isokinetic muscle strength PROs (KOOS, Euro‐QoL‐5D)	None
Lützner et al. (2023)	Germany	TKA	Treatment	Coated implant	Active (standard implant)	PROs (OKS, SF36, UCLA Activity Scale)	Serum cytokine levels
Marinova et al. (2023)	Australia	TKA	Treatment	Cryocompression Game Ready™	Active (usual care)	PROs (OKS, FJS, KOOS) Postoperative complications	None
Nam et al. (2023)	South Korea	TKA	Treatment	Standard preoperative education and additional module on realistic expectations following TKA	Active (standard preoperative education)	Patient satisfaction	PROs (Short Form‐36) Crepitus score ROM
Nørgaard Linde Karina et al. (2022)	Denmark	TKA	Treatment	Cementless tibial components	Active (cemented tibial components)	Component migration (radiostereometry) Changes in bone mineral density Biochemical markers of bone turnover	PROs (KSS) Pain (VAS) Complications
Petersen et al. (2022)	Denmark	TKA	Treatment	Implant with medial congruent anatomic bearing design	Active (implant with standard symmetrical cruciate retaining bearing design)	PROs (OKS, FJS, KOOS) Postoperative complications	None
Saccomanno et al. (2022)	Italy	Traumatic anterior shoulder instability treatment	Treatment	PEEK knotless	Active (knotted biodegradable suture anchors)	PROs (DASH)	PROs (Work‐DASH, Sport‐DASH) Rowe score Recurrence rate of dislocation Re‐operation rate Intraoperative complications Postoperative complications
Schittek et al. (2022)	Austria	TKA	Treatment	Periarticular infiltration anaesthesia	Active (ultrasound‐guided regional anaesthesia with femoral and popliteal block)	Opioid consumption (OME) Pain (NRS) Complications	None
Snow et al. (2023)	United Kingdom	Arthroscopic rotator cuff repair	Treatment	Acellular dermal patch	Active (non‐acellular dermal patch)	Rotator cuff retear (MRI) Sugaya classification	PROs (WORC, ASES, Quick‐DASH)
Sørensen et al. (2023)	Denmark	ACL reconstruction	Treatment	Combined ACL reconstruction revision with anterior‐lateral ligament reconstruction	Active (ACL reconstruction revision)	PROs (KNEES‐ACL, KOOS, TAS) Knee laxity (rollimeter test, pivot shift test, manual Lachman test)	None
Straume‐Naesheim et al. (2022)	Norway	MPFL reconstruction	Treatment	Isolated MPFL reconstruction	Active (active rehabilitation)	PROs (KOOS, Kujala score, Lysholm score, Noyes sports activity rating scale, modified Cincinnati knee rating system) Pain (VAS) ROM Apprehension to lateralisation of the patella	None
Swamy et al. (2023)	India	TKA	Treatment	Accelerometer‐based portable navigation	Active (computer‐assisted navigation)	Tourniquet time Blood loss Radiological outcomes	Flexion deformity ROM PROs (KSS, OKS)
Ulivi et al. (2023)	Italy	Knee osteoarthritis	Treatment	Arthroscopic debridement with microfragmented adipose tissue	Active (arthroscopic debridement)	Pain (VAS) PROs (KOOS, KSS, WOMACSF‐12,) Radiological outcomes T2 mapping analysis Biochemical analysis	None
Yang et al. (2023)	China	Arthroscopy skill acquisition	Other	One‐day intervals	Active Two‐day intervals One‐week intervals	Learning effect collected by the stimulator Long‐term skill retention (post‐test)	Short‐term skill retention (post‐test)
Zhang et al. (2023)	China	Femoral nerve block	Treatment	Ropivacaine 0.2% concentration	Active (ropivacaine 0.1% concentration	Quadriceps strength Pain (NRS)	Knee mobility (knee extension test) Adverse events Self‐reported patients satisfaction Length of hospital stay

Abbreviations: ACL, anterior cruciate ligament; AKSS, American Knee Society Score; ASES, American Shoulder and Elbow Score; DASH, Disabilities of the Arm, Shoulder and Hand; DVT, deep vein thrombosis; ERAS, Enhanced Recovery after Surgery; FJS‐12, Forgotten Joint Score‐12; IKDC, International Knee Documentation Committee (Subjective Score); KNEES‐ACL, Knee Numeric‐Entity Evaluation Score; KOOS‐QoL, Knee Injury, and Osteoarthritis Outcome Score for Quality of Life; KOOS‐S, Knee Injury, and Osteoarthritis Outcome Score for Symptoms; KSS, Knee Society Score; LEAS, Levels of Emotional Awareness Scale; MPFL, medial patellofemoral ligament; NRS, Numerating Rating Scale; OKS, Oxford Knee Score; OME, oral morphine equivalents; PCL, posterior cruciate ligament; PROs, patient‐reported outcomes; ROM, range of motion; SF‐12, Short Form Health Survey; STAI‐6, 6‐Item State Anxiety Scale; TAS, Tegner Activity Score; TKA, total knee arthroplasty; VAS, visual analogue scale; WOMAC, Western Ontario and McMaster Universities Arthritis Index; WORC, Western Ontario Rotator Cuff Index.

**Figure 2:**
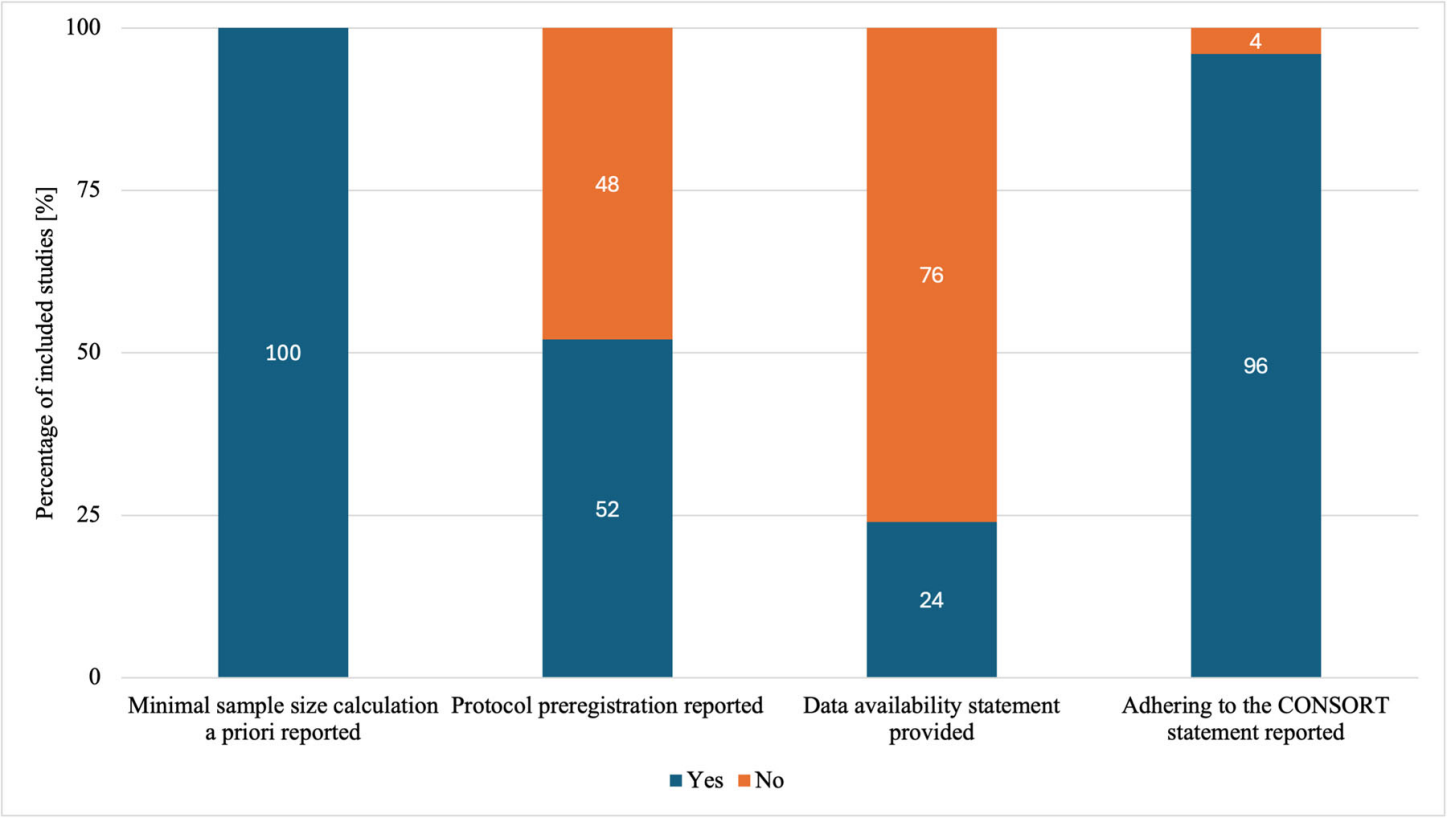
Reporting quality indicators (sample‐specific information, data transparency, adherence to reporting guidelines) in the included studies.

In Table 2, reporting quality indicators in the included studies were presented. A visual representation of the ‘yes’ and ‘no’ answers can be found in Figure 2. All the studies included in the analysis had a predetermined minimum sample size, as indicated in Figure 2. Additionally, as depicted in the same figure, 52% of the studies indicated that their study protocols were preregistered, while only 24% included statements about data availability. As presented in Table 2, among the five out of six studies that provided data availability statements, it was mentioned that the data could be made available upon a (reasonable) request to the corresponding author. One study simply stated that data can be provided. Adherence to the CONSORT statement was reported in 96% of the studies (Figure 2). Only one study did not adhere to CONSORT or any other recognised guidance for reporting RCTs (Table 2).

**Table 2:** Reporting quality indicators in included studies.

Authors and year	Sample‐specific information		Protocol preregistration	Data transparency		Adherence to reporting guidelines	
	Number of (analysed) participants	Minimal sample size calculation a priori reported	Protocol preregistration reported	Data availability statement provided	If yes, specific information included	Adhering to the CONSORT statement reported	Adhering to any other recognised guidance for reporting RCTs than the CONSORT statement
Bollars et al. (2023)	Total *n* = 52 Robotic‐assisted TKA *n* = 26 Conventional jig‐based instrumented TKA *n* = 26	Yes	Yes	No	Not applicable	Yes	Not applicable
Budhiparama et al. (2023)	Total *n* = 33 PCL preserved *n* = 33 PCL resected *n* = 33	Yes	No	Yes	*The data that support the findings of this study are available from the corresponding author upon reasonable request*.	Yes	Not applicable
Chu et al. (2022)	Total *n* = 644 P‐PRP *n* = 322 Saline *n* = 322	Yes	No	No	Not applicable	Yes	Not applicable
Degen et al. (2023)	Total *n* = 132 Group 1 *n* = 33 Group 2 *n* = 34 Group 3 *n* = 33 Group 4 *n* = 32	Yes	Yes	Yes	*The data of this. study are available from the corresponding author upon request*.	Yes	Not applicable
Demirci et al. (2023)	Total *n* = 50 Audio‐visual distraction *n* = 25 Audio distraction *n* = 25	Yes	No	No	Not applicable	Yes	Not applicable
Farhan‐Alanie et al. (2023)	Total *n* = 78* Navigated TKA *n* = 41* Conventional TKA *n* = 37* *Completed 10 years follow‐up	Yes	No	No	Not applicable	Yes	Not applicable
Goicoechea et al. (2023)	Total *n* = 198 Lateral retinacular release *n* = 98 No lateral retinacular release *n* = 100	Yes	No	No	Not applicable	Yes	Not applicable
Hoburg Arnd et al. (2022)	Total *n* = 102 ACI *n* = 52 Microfracture *n* = 50	Yes	Yes	No	Not applicable	Yes	Not applicable
Kalaai et al. (2023)	Total *n* = 38 Mobile bearing TKA *n* = 20 Fixed bearing TKA *n* = 18	Yes	No	No	Not applicable	No	No
Lavoie et al. (2023)	Total *n* = 74* Bicruciate‐retaining prosthesis *n* = 37* Posterior‐stabilised prosthesis *n* = 37* *Completed 5 years follow‐up	Yes	Yes	No	Not applicable	Yes	Not applicable
Lee et al. (2023)	Total *n* = 173 Limited tourniquet *n* = 43 Conventional tourniquet *n* = 47	Yes	Yes	No	Not applicable	Yes	Not applicable
Lützner et al. (2023)	Total *n* = 105 Coated implant TKA *n* = 55 Standard implant TKA *n* = 50 *Completed 5 years follow‐up	Yes	Yes	No	Not applicable	Yes	Not applicable
Marinova et al. (2023)	Total *n* = 72 Cryocompression Game Ready™ *n* = 36 Usual care *n* = 36	Yes	No	No	Not applicable	Yes	Not applicable
Nam et al. (2023)	Total *n* = 158 Standard preoperative education and additional module on realistic expectations following TKA *n* = 78 Standard preoperative education *n* = 80	Yes	No	Yes	*The data that support the findings of this study are available upon reasonable request*.	Yes	Not applicable
Nørgaard Linde et al. (2022)	Total *n* = 51* Cementless TKA *n* = 26* Cemented TKA n = 25* *Completed 2 years follow‐up	Yes	Yes	No	Not applicable	Yes	Not applicable
Petersen et al. (2022)	Total *n* = 64 Implant with medial congruent anatomic bearing design *n* = 33 Implant with standard symmetrical cruciate retaining bearing design *n* = 31	Yes	Yes	No	Not applicable	Yes	Not applicable
Saccomanno et al. (2022)	Total *n* = 71 PEEK knotless *n* = 34 Knotted biodegradable suture anchors *n* = 37	Yes	No	No	Not applicable	Yes	Not applicable
Schittek et al. (2022)	Total *n* = 50 Periarticular infiltration anaesthesia *n* = 25 Ultrasound‐guided regional anaesthesia with femoral and popliteal block *n* = 25	Yes	Yes	No	Not applicable	Yes	Not applicable
Snow et al. (2023)	Total *n* = 40 Acellular dermal patch *n* = 20 Non‐acellular dermal patch *n* = 20	Yes	No	Yes	*The data supporting the findings (…) can be made available from the corresponding author upon request*.	Yes	Not applicable
Sørensen et al. (2023)	Total *n* = 89* ACL revision + ALL *n* = 39 Isolated ACL *n* = 50 *Completed 2 years follow‐up	Yes	Yes	Yes	*Data can be provided*.	Yes	Not applicable
Straume‐Naesheim et al. (2022)	Total *n* = 59 MPFL reconstruction and active rehabilitation *n* = 30 Active rehabilitation only *n* = 29	Yes	Yes	No	Not applicable	Yes	Not applicable
Swamy et al. (2023)	Total *n* = 47 Accelerometer‐based portable navigation *n* = 24 Computer‐assisted navigation *n* = 23	Yes	Yes	No	Not applicable	Yes	Not applicable
Ulivi et al. (2023)	Total *n* = 56 Arthroscopic debridement with microfragmented adipose tissue *n* = 28 Arthroscopic debridement *n* = 28	Yes	No	No	Not applicable	Yes	Not applicable
Yang et al. (2023)	Total *n* = 30 One‐day intervals *n* = 10 Two‐day intervals *n* = 10 One‐week intervals *n* = 10	Yes	No	Yes	*The data that support the findings of this study are available from the corresponding author upon reasonable request*.	Yes	Not applicable
Zhang et al. (2023)	Total *n* = 83 Ropivacaine 0.2% concentration *n* = 41 Ropivacaine 0.1% concentration *n* = 42	Yes	Yes	No	Not applicable	Yes	Not applicable

Abbreviations: ACI, autologous chondrocyte implantation; ACL anterior cruciate ligament; AD, arthroscopic debridement; ALL, anterolateral ligament; MPFL, medial patellofemoral ligament; *n*, number of participants; P‐PRP, pure platet‐rich plasma; PCL, posterior cruciate ligament; TKA, total knee arthroplasty.

The results of the critical appraisal of individual sources of evidence are presented in Table 3.

**Table 3:** The results of the critical appraisal of the included studies (green: yes; red: no; yellow: unclear).

Authors and year	Q1	Q2	Q3	Q4	Q5	Q6	Q7	Q8	Q9	Q10	Q11	Q12	Q13
Bollars et al. (2023)	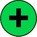	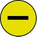	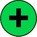	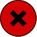	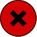	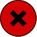	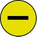	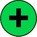	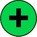	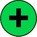	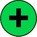	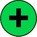	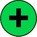
Budhiparama et al. (2023)	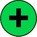	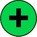	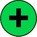	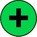	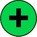	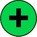	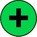	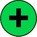	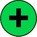	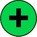	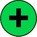	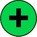	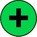
Chu et al. (2022)	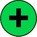	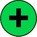	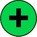	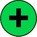	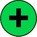	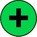	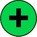	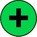	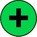	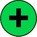	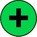	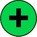	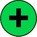
Degen et al. (2023)	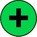	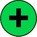	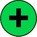	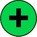	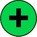	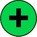	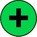	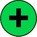	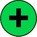	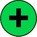	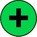	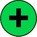	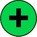
Demirci et al. (2023)	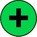	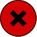	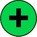	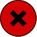	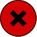	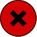	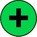	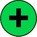	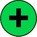	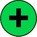	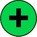	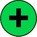	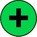
Farhan‐Alanie et al. (2022)	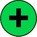	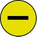	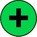	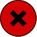	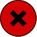	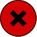	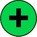	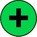	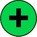	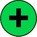	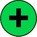	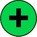	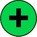
Goicoechea et al. (2023)	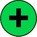	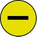	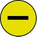	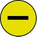	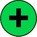	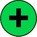	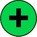	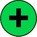	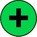	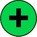	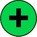	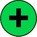	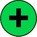
Hoburg et al. (2022)	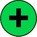	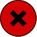	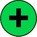	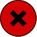	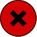	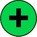	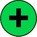	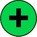	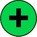	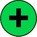	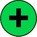	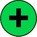	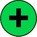
Kalaai et al. (2023)	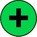	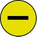	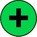	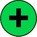	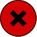	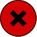	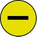	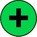	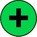	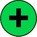	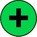	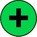	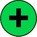
Lavoie et al. (2022)	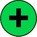	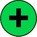	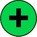	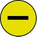	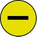	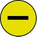	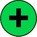	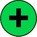	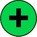	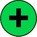	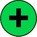	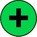	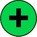
Lee et al. (2022)	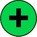	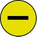	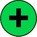	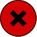	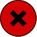	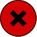	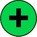	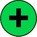	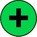	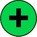	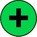	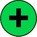	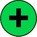
Lützner et al. (2022)	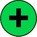	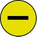	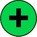	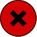	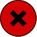	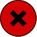	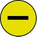	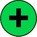	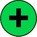	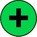	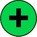	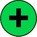	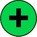
Marinova et al. (2023)	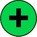	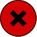	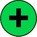	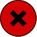	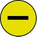	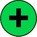	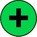	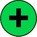	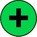	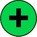	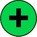	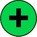	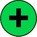
Nam et al. (2023)	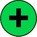	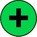	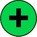	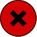	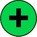	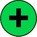	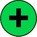	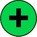	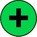	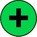	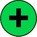	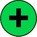	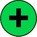
Nørgaard Linde et al. (2022)	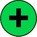	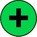	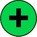	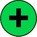	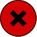	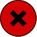	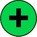	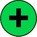	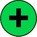	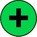	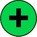	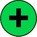	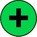
Petersen et al. (2022)	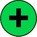	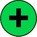	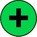	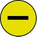	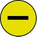	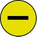	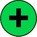	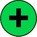	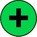	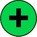	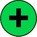	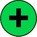	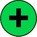
Saccomanno et al. (2022)	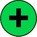	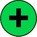	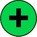	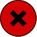	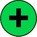	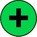	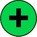	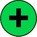	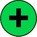	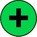	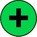	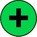	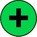
Schittek et al. (2022)	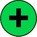	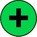	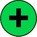	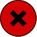	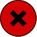	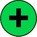	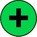	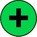	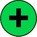	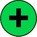	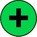	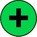	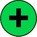
Snow et al. (2023)	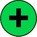	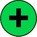	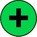	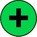	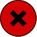	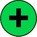	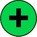	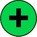	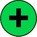	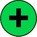	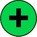	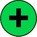	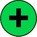
Sørensen et al. (2023)	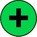	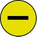	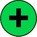	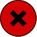	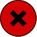	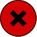	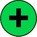	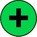	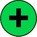	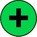	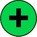	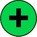	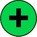
Straume‐Næsheim et al. (2022)	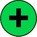	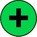	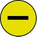	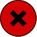	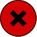	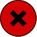	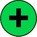	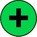	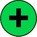	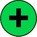	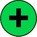	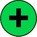	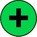
Swamy et al. (2022)	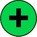	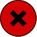	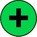	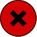	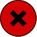	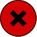	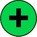	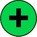	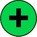	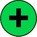	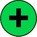	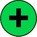	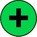
Ulivi et al. (2022)	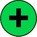	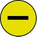	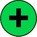	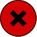	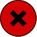	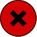	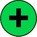	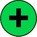	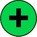	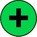	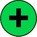	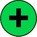	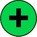
Yang et al. (2023)	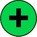	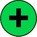	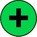	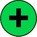	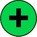	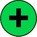	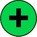	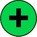	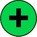	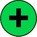	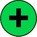	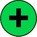	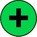
Zhang et al. (2023)	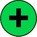	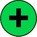	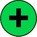	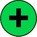	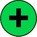	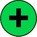	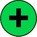	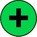	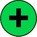	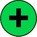	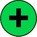	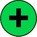	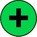

*Note*: Q1 Was true randomisation used for assignment of participants to treatment groups? Q2 Was allocation to treatment groups concealed? Q3 Were treatment groups similar at the baseline? Q4 Were participants blind to treatment assignment? Q5 Were those delivering the treatment blind to treatment assignment? Q6 Were outcome assessors blind to treatment assignment? Q7 Were treatment groups treated identically other than the intervention of interest? Q8 Was follow‐up complete, and if not, were differences between groups in terms of their follow‐up adequately described and analysed? Q9 Were participants analysed in the groups to which they were randomised? Q10 Were outcomes measured in the same way for treatment groups? Q11 Were outcomes measured in a reliable way? Q12 Was appropriate statistical analysis used? Q13 Was the trial design appropriate and any deviations from the standard RCT design (individual randomisation, parallel groups) accounted for in the conduct and analysis of the trial?

## DISCUSSION

The results of this scoping review largely support the initial hypothesis that the majority of RCTs published in *KSSTA*, the official journal of *ESSKA*, between 2022 and 2023 adhered to key reporting guidelines, including CONSORT. However, while adherence to these guidelines was observed in 96% of the included studies, gaps in other critical areas of reporting were evident. Specifically, only 52% of studies reported protocol preregistration, and 24% provided data availability statements, highlighting deficiencies in transparency and reproducibility. These findings suggest that while methodological rigour in reporting some aspects—such as sample size calculation—was strong, improvements are needed in promoting data transparency and ensuring the preregistration of study protocols.

As mentioned, articles focusing on enhancing evaluation standards for clinical studies in orthopaedic and sports medicine have been published since the end of 2022. These include providing sample‐specific information, ensuring data transparency and adhering to reporting guidelines [49].

It has to be highlighted, that all the studies included in the present review had a predetermined minimum sample size. Calculating the minimum needed sample size is critical because it ensures statistical power, minimises inconclusive research, addresses ethical considerations and reduces so‐called research waste [49]. Failure to perform this calculation can lead to a study being underpowered, meaning it might miss true effects due to a lack of data, or overpowered, which can result in unnecessary complexity and resource usage. In their editorial, Madjarova et al. comprehensively addressed the knowledge‐to‐practice gap around statistical power [35]. They demonstrated how power is affected by four factors—*p* value, effect size, sample size and variance. The editorial discussed the advantages and disadvantages of a priori versus post hoc power analyses. The editorial provided researchers with suggested resources for sample size calculation and the necessary context to understand statistical fragility [35]. The issue of calculating the minimum needed sample a priori has been a matter of concern not only in the light of RCTs but also in the context of reliability studies [24].

Another way of preventing research waste is to rely on earlier studies, which form the basis for evidence‐based research and are extensively explained by Prill et al. [52]. Redundant or low‐value studies due to issues like lack of novelty or weak methodology are growing concerns in the research field. This highlights the importance of transparent justification for new studies and the need for robust research methods in orthopaedics, sports medicine and rehabilitation. Systematic reviews are crucial in synthesising existing evidence, identifying knowledge gaps, and guiding future research. Clinicians can apply these findings to practice, while researchers use them to inform research priorities. Recognising the value of sound methodology, the official journal of *ESSKA* also emphasises educating researchers on systematic reviews and meta‐analyses [20, 26, 27, 48].

The contentious issue of open and transparent research, which has been a subject of great debate, has been tackled by Hansford et al. [16]. Addressing this urgent need to clarify the principles of study protocols and preregistration in orthopaedics, sports medicine and rehabilitation, Prill et al. [47]. Unfortunately, the present review uncovered significant gaps in data transparency in the included studies. Only half of the studies reported protocol preregistration, and just a quarter included data availability statements. This underscores the need for greater emphasis on open science practices, enhanced transparency, and reproducibility in research. What is also critical is that none of the studies that provided data availability statements provided data in public repositories. Instead, they offered data upon request from the corresponding author. While offering data on request is a step toward transparency, publicly accessible data in repositories allows for greater reproducibility, peer validation, and secondary analysis. In the context of RCTs, where robust evidence is critical for clinical decision‐making, providing data in public repositories ensures that findings can be independently verified. Public repositories also facilitate better compliance with open science principles, making research more transparent and fostering trust within the scientific community [49]. This highlights an important area needing improvement, as public data sharing is increasingly expected to advance scientific integrity and transparency.

In recent years, there has been a growing emphasis on the significance of transparent reporting and adherence to guidelines such as the CONSORT to guarantee that RCTs are entirely reproducible, unbiased, and useful for other researchers and clinicians. Substandard reporting practices, lack of transparency in data availability and failure to comply with reporting standards can undermine the usefulness of even well‐conducted studies. The high adherence to the CONSORT statement, reported in 96% of the included studies in the present review, demonstrates a solid commitment to standardised reporting practices. Given the stringent requirements of conducting an RCT, using an appropriate reporting tool is essential [50]. One of the most widely utilised documents for reporting RCTs is the CONSORT 2010 statement, which includes a checklist and a flow diagram [57]. The checklist comprises 25 items detailing how the trial was designed, analysed, and interpreted, while the flow diagram illustrates the progress of participants from enrolment to final analysis. The CONSORT 2010 Explanation and Elaboration document provides further clarification of the checklist [39]. It should be noted that CONSORT 2010 primarily addresses two‐group parallel designs, with extensions developed for other study designs.

All the included studies adequately addressed the so‐called statistical conclusion validity. However, internal validity was less consistently addressed across the studies, indicating potential weaknesses in controlling for biases and ensuring the reliability of causal relationships.

In all the included studies, true randomisation was used to assign participants to treatment groups. However, the main issue seems to lie with the blinding procedures, which can be somewhat challenging in orthopaedics, traumatology, and sports medicine. In 36% of the included studies, the treatment assignment for participants, those delivering the treatment, and outcome assessors were not blinded. For example, Bollars et al. noted that it was impossible to blind the patients and the surgeon, as they knew whether imageless handheld robotic‐assisted TKA or conventional jig‐based instrumented TKA was used [4]. Similarly, Demirci et al. explained that blinding was not feasible because the operative team and the patient were aware of the assigned intervention—precisely, audio‐visual distraction with video glasses or audio distraction—after randomisation [12]. Sørensen et al. clarified that the patients were not blinded to the procedure, as the combined anterior cruciate ligament reconstruction revision with anterior‐lateral ligament reconstruction involved two extra incisions on the lateral side, which also made it impossible to blind the investigator regarding group assignments [59]. Lützner et al. indicated only that the study was not blinded [33]. Ulivi et al. provided a more detailed explanation, stating that the primary limitation was the lack of blinding for patients and assessors, stemming from ethical concerns over performing an additional procedure. They also noted that follow‐up assessments were conducted via phone interviews, which limited the availability of clinician‐reported scores and imaging evaluations at the time. Furthermore, they highlighted an imbalance in baseline scores and conditions between the two groups due to the randomisation process, which posed further limitations. While the authors used appropriate statistical methods to address these differences, they acknowledged the potential for bias in outcomes, which may have influenced the control of confounding variables [62].

In orthopaedics and traumatology, where surgical interventions are common, blinding patients and those administering the treatment can be particularly challenging. For instance, the physical nature of surgeries often makes it impossible to conceal the type of procedure from patients or surgeons, as sham surgeries would be unethical. However, every effort should still be made to blind assessors who evaluate patient outcomes to minimise bias. In some cases, specific aspects of an intervention may not be blinded due to ethical concerns or practical limitations, such as the visibility of surgical scars or the inability to mask post‐operative care. Nevertheless, blinding outcome assessors remains a crucial strategy to preserve the integrity of the trial results. In the study by Marinova et al., measurements were initially collected in the hospital by the patient's treating physiotherapist, who was not blinded to the treatment. Follow‐up measurements were taken by a blinded research assistant during subsequent appointments [37]. In the study by Schittek et al., patients and treating physicians were aware of the group assignment. However, data collection was performed by physicians blinded to the patient's anaesthetic treatment [56]. Snow et al. stated that their study was double‐blinded. Blinding was implemented for patients and treatment providers, but no specific information about the outcome assessors was provided [58].

Still, Budhiparama et al., Chu et al., Degen et al., Yang et al. and Zhang et al. correctly addressed all items of internal validity [5, 6, 11, 66, 67]. For example, in the Budhiparama et al. study, the main surgeon randomly drew a sealed envelope from a box before the surgery. The envelope contained the name of the intervention, precisely the name of the side on which the posterior cruciate ligament would be resected during bilateral TKA. The patients were not aware of which knee the posterior cruciate ligament was resected. The assessments were performed by a single orthopaedic surgeon, who was not a part of the main surgical team and was not aware of the patients' group allocations until the end of the study [5]. In the Degen et al. study, surgeons, data collectors, and patients were blinded to treatment allocation. To ensure this, pharmacy personnel prepared and masked both active medications and placebo pills in a manner that made them indistinguishable in appearance. Patients in the intervention groups received their respective treatments, while those in the control and alternative intervention groups were given placebo pills at identical intervals to maintain blinding throughout the preoperative and postoperative phases [11].

The review indicated that the primary outcomes in the included studies have been assessed using a wide variety of objective and subjective methods, including patient‐reported outcome measures PROMs. It is important to remember that the selection of the primary outcome, on which the study's purpose, methodology, and conclusions are based, should be made with great caution. Particular caution must be preserved when the primary outcome is PROM, which has been raised and widely explained by Krogsgaard et al. [23]. The challenges of using PROMs and the controversies around them, including using them as primary outcomes, have also been comprehensively presented by Królikowska et al. [25].

Still, the mentioned series of articles in the *ESSKA* official journal covered more than just the essential three components of rigorous methodology. There has also been a significant focus on incorporating precise statistical data into orthopaedics and sports medicine studies. An important issue that has been brought to attention is the incorrect implementation of regression analyses, despite multivariable regression being a fundamental tool that drives observational research in orthopaedic surgery. Varady et al. provided a comprehensive overview of regression analyses, addressing common areas of confusion. The aim was to enhance statistical literacy in orthopaedic research by clarifying the use and interpretation of multivariable analyses [63].

Consecutively, Madjarova et al. highlighted common misconceptions about *p* values and the harmful consequences of misinterpreting this widely used statistical measure. They aimed to provide clarity on the concept of statistical significance for those seeking to improve their understanding of statistics in orthopaedic research [36]. Continuing the topic of statistically significant difference, Ostojic et al. confronted it with a clinically relevant difference [45]. They emphasised that a statistically significant difference is a mathematical term that indicates the unlikelihood of a difference occurring by chance. This significance is amplified in studies with large sample sizes, but it does not necessarily mean it will have a clinical impact on an individual. The editorial underscored integrating statistical analysis and clinical judgement to better understand the minimal clinically important difference (MCID). This approach is a valuable tool for bridging the gap between clinical and statistical significance, as the authors called it [3]. Mabrouk et al. significantly explored the topic of MCID and expanded the clinical significance of statistically significant results, explaining the concept of the patient‐acceptable symptom state [34]. Also, the survival analyses and their applications in orthopaedics were broadly explained by Pruneski et al. [53].

Medical journals play a crucial role in rapidly addressing emerging developments, educating their readers on new advancements, and keeping them updated on best scientific research practices. For example, the *KSSTA* journal has recently highlighted topics such as developing and using deep learning models for orthopaedic surgeons, the application of machine learning in healthcare, the potential of artificial intelligence in improving surgical outcomes, and the ethical considerations surrounding data privacy and registry creation. It has also included a series of articles on research methodologies, including but not limited to statistics and guidelines for research [7, 13, 22, 28, 31, 38, 41, 42, 43, 54, 64]. These efforts help keep practitioners informed on innovative technologies and their practical implications in orthopaedics. Topics related to improving orthopaedic scientific communication, such as the introduction of infographics and the role of social media, have also been discussed [8, 65]. The journal also addressed controversial emerging topics, such as the usage of Chat GPT [21, 44].

When discussing the improvement and maintenance of the highest quality of reporting in medical journals, it is essential to mention the role of reviewers. Reviewers play a vital role as gatekeepers to ensure that published research meets the highest standards of accuracy, transparency and methodological rigour. Their decisions directly impact the quality and credibility of medical literature, making their role critical in advancing reliable and trustworthy science. Reviewers are typically not provided with formal training on conducting a review or peer‐review process. Furthermore, there is a common belief that experienced authors are well‐suited to be reviewers, but this assumption may only sometimes align with reality. Therefore, it is essential to recognise that being an experienced author does not automatically equate to being an effective reviewer. Hence, any education on conducting a meaningful review also holds immense value and deserves appreciation [18].

## CONCLUSIONS

This scoping review identified that, while most recently published RCTs in *KSSTA* adhered to CONSORT guidelines, significant gaps were found in reporting protocol preregistration and data availability statements. Although all studies reported sample size calculations, transparency in data sharing remains limited. Future efforts should focus on improving preregistration practices and data transparency to enhance the overall quality and reproducibility of research in this field. Additionally, all blinding options, including assessor and statistician blinding, should be rigorously considered when designing RCTs in orthopaedics.

## AUTHOR CONTRIBUTIONS

*Conception*: Aleksandra Królikowska and Robert Prill. *Design*: Aleksandra Królikowska, Paweł Reichert and Robert Prill. *Preparing the draft of the manuscript*: Aleksandra Królikowska, Natalia Urban, Marcin Lech and Nikolai Ramadanov. *Critical revision of the manuscript for important intellectual content*: Paweł Reichert, Mahmut Enes Kayaalp and Robert Prill. *Approval of the version to be published*: Aleksandra Królikowska, Natalia Urban, Marcin Lech, Paweł Reichert, Nikolai Ramadanov, Mahmut Enes Kayaalp and Robert Prill. *Agreement to be accountable for all aspects of the work in ensuring that questions related to the accuracy or integrity of any part of the work are appropriately investigated and resolved*: Aleksandra Królikowska, Natalia Urban, Marcin Lech, Paweł Reichert, Nikolai Ramadanov, Mahmut Enes Kayaalp and Robert Prill.

## CONFLICT OF INTEREST STATEMENT

The authors declare no conflicts of interest.

## ETHICS STATEMENT

Ethical approval does not apply to this study, as it is a scoping review based on previously published studies and does not involve collecting primary data from human participants.

## Data Availability

The data supporting this scoping review are derived from publicly available articles. These sources can be accessed through the databases and journals listed in the Methods section. No additional data sets were generated or analysed during the review.
